# Impact of long-term fasting on liver and abdominal adipose tissues monitored by quantitative chemical-shift encoded MRI

**DOI:** 10.3389/fnut.2026.1868854

**Published:** 2026-09-07

**Authors:** Magalie Viallon, Benjamin Leporq, Helène Ratiney, Françoise Wilhelmi de Toledo, Robin Mesnage, Pierre Croisille

**Affiliations:** 1 Univ Lyon, UJM-Saint-Etienne, INSA, Saint-Etienne, France; 2 Department of Radiology, University Hospital Saint-Etienne, Saint-Etienne, France; 3 Univ Lyon, INSA-Lyon, Université Claude Bernard Lyon 1, UJM-Saint Etienne, CNRS, Inserm, Lyon, France; 4 Buchinger Wilhelmi Clinic, Überlingen, Germany; 5 King’s College London, Department of Nutritional Sciences, School of Life Course Sciences, Faculty of Life Sciences and Medicine, London, United Kingdom

**Keywords:** abdominal fat, fasting, lipid metabolism, liver, MRI, quantitative imaging, nutritional intervention

## Abstract

**Background and Aims:**

This study used quantitative Magnetic Resonance Imaging (MRI) to explore lipid metabolism changes during a 12-day fasting intervention in the liver and abdominal adipose tissue (AT).

**Methods:**

MRI was performed on 32 participants in the GENESIS clinical trial. This prospective, monocentric, single-arm interventional study evaluated body composition changes at four time points: before, at the end of 12 fasting days, and at 1 and 4 months after refeeding. The MRI protocol included a whole-body multi-step Dixon 3D gradient-echo sequence and a 3D chemical-shift encoded (CSE) pulse sequence to quantify different MRI metrics, such as proton density fat content (PDFF), fatty acid composition [proportion of saturated, monounsaturated, and polyunsaturated fatty acids (FA)], and T_2_*. Automated segmentations extracted quantitative metrics from organs, including the liver and visceral and subcutaneous fat, and measured their volumes.

**Findings:**

Fasting strongly reduced the liver volume (*p* < 0001) beyond the classical reduction in weight and blood triglyceride levels. We observed marked temporal variations in subcutaneous and visceral AT volumes, with a noteworthy continuous downward visceral fat reduction up to 4 months after fasting. Different trends were observed in liver fat content: it increased temporarily in healthy subjects, but decreased in those with liver steatosis. The increase reversed after 1 month, whereas the decrease in persistence varied during follow-up. The proportion of monounsaturated FA in AT decreased, whereas no change was observed for saturated and polyunsaturated FA during fasting.

**Interpretation:**

CSE-MRI enables fast, injection-free, and quantitative monitoring of abdominal organs during fasting. Our results suggest that long-term fasting can help prevent metabolic dysfunction-associated fatty liver disease by reducing liver fat and promoting a lasting reduction in high-risk visceral fat.

## Highlights

- Strong reduction in liver volume after fasting.
- Marked temporal variations of subcutaneous and visceral AT volumes with sustainable visceral fat reduction until four months after fasting.
- Adaptation of liver fat content to fasting with an increase in fat in normal subjects sustains organ fuel needs, and a beneficial excess fat decrease in steatosis in clinically healthy subjects.

## Introduction

In animals, such as birds, liver steatosis is a physiological adaptive response that allows the liver to buffer excess fatty acids and ensure survival during the migration fasting period (1, 2). In humans, fat accumulation in the liver is not followed by periods of fasting and intensive physical activity, whereas excessive and chronic steatosis disrupts normal liver metabolism and triggers metabolic stress. Liver steatosis is now recognized as the hallmark of metabolic dysfunction-associated fatty liver disease (MAFLD), whose global incidence has been increasing, reaching close to 25% of the global population in Western countries, including children (3). This condition, which is defined by the pathological accumulation of lipids in the liver independent of alcohol consumption (4), can progress to metabolic dysfunction-associated steatohepatitis (MASH) and eventually to fibrosis, cirrhosis, liver failure, and hepatocellular carcinoma (5). The increasing prevalence of MAFLD is related to unhealthy lifestyles, particularly excessive intake of glucose, fructose, and saturated fat, and insufficient physical activity (6).

The first-line treatment approach involves lifestyle modifications, including dietary changes and increased physical activity to promote weight loss, as there have been no approved pharmacological agents for MAFLD and MASH until recently. Research indicates that losing 5% of body weight can help reduce hepatic steatosis, while a weight loss of more than 7% is linked to improvements in the MASH score (7). Additionally, achieving a weight loss of 10% or more is linked to significant resolution of MASH and regression of fibrosis (8).

Caloric restriction and fasting are emerging as promising methods rooted in human evolution, where periodic fasting for fat mobilization is common due to seasonal food scarcity. A decrease in calorie intake leads to a rapid reversal of hepatic insulin resistance and MAFLD (9). Restoration of natural circadian rhythms and intermittent fasting favoring insulin sensitivity are among the most efficient methods for treating MAFLD (10), and normalization of the fatty liver index as a surrogate parameter of MAFLD following prolonged fasting (11).

Based on a preliminary study showing the unique potential of Magnetic Resonance Imaging (MRI) quantitative metrics to explore tissue composition dynamics during prolonged fasting (12), the GENESIS study aimed to assess the longitudinal effects of a 12-day fasting intervention on multiple organs in a representative population of healthy men and women (13).

In this prospective longitudinal study, we used quantitative magnetic resonance imaging (MRI) to investigate changes occurring in liver, subcutaneous, and visceral adipose tissues from baseline, at the end of a 12-deay fast, and 1 and 4 months after food reintroduction.

## Materials and methods

### Participants and fasting protocol

This prospective, monocentric, single-arm interventional study enrolled between September 2021 and March 2022, 16 men and 16 women (44 ± 14 years; 26.2 ± 0.9 kg/m^2^; matching eligibility criteria listed in Supplementary Table S1) who fasted for 12 days and maintained up to 3 h of daily low-intensity physical activity. The fasting protocol and the sample size calculation are detailed in the GENESIS (lonG tErm FastiNg multi-systEm adaptationS In humanS) study protocol article (13). During the fasting period, the energy intake was limited to 200–250 kcal/day (10), which was achieved with 0.25 L freshly squeezed, organic fruit juice at noon, 0.25 L vegetable soup in the evening, and 20 g honey. Participants will be asked to drink a minimum of 2 L of water or non-caloric, caffeine-free herbal tea purchased by certified organic companies. Every second fasting day, the colon is emptied by an enema or laxative. On the last fasting day, plant-based organic food will be progressively reintroduced over 3–4 consecutive days (800–1,600 kcal/day). The fasting intervention was supervised by medical doctors and nurses. An accompanying program consisting of physical exercise, mindfulness, meditation, and group interactions was provided. The training program included daily outdoor walks (1.5 h), moderate-intensity fitness exercises, and free access to a gym and swimming pool. As part of the evaluation, participants underwent MRI scans to evaluate body composition and main organ changes at four time points: before (D-1), at the end of 12 fasting days (D + 12), one (M1) and four (M4) months after the end of fasting and food reintroduction. The Baden-Württemberg Medical Council approved this study (F-2021-075, July 26, 2021), and the study was registered at ClinicalTrials.gov (NCT05031598). Signed informed written consent was obtained from all participants.

### MRI acquisition

For body composition measurements (adipose tissue compartments, muscle volumes, and muscle tissue composition, accurate measurements of visceral adipose tissue, abdominal subcutaneous adipose tissue; see Supplementary material), the participants were imaged in the supine position with the arms along the sides on a 3 T Prisma (Siemens Healthineers, Erlangen, Germany). Reconstruction of the Dixon water and Dixon fat images was performed using the integrated scanner software. A higher resolution VIBE (Volumetric Interpolated Breath-hold Examination) sequences in transversal plane was acquired for volumetry of the liver (VIBE e-Dixon sequence: repetition time/echo time (TR/TE) = 3.97/1.29 ms, resolution 1.2 × 1.2 × 3 mm, flip angle = 9°, 80 slices, acceleration factor 2 × 2 caipirinha, automatic segmentation routine of Siemens). Reconstruction of Dixon water and Dixon fat images was performed in line with the integrated scanner software, as well as automatic liver segmentation. Examples of whole-body reconstructed water and fat images are shown in Supplementary Figure S2, as well as evolution and changes over time on one participant at all important time points of observation settled in the GENESIS study.

Breath-hold transverse MRI acquisitions were added at the abdominal stage for specific biomarker extraction, that is, simultaneous quantification of fat content, fatty acid composition, and T_2_* in the abdomen (from the liver dome to L5-S1), using 3D spoiled-multiple echo gradient echo sequences with a flyback readout gradient (14). The main MR parameters for the sequences used are listed in Supplementary Figure S2, S3.

### Post processing

We calculated the PDFF, T_2_*, and fatty acid composition using the method validated in (14) and implemented in MATLAB R2022b (The MathWorks Inc., Natick, MA, USA). Briefly, a phase correction algorithm was used to unwrap and correct the native phase images for the zero- and first-order phases and rebuild the B0- demodulated real part images. Then, using a model of fat ^1^H MR spectrum integrating eight components, the number of double bonds (ndb) and methylene-interrupted double bonds (nmidb) was derived voxel-by-voxel by a stepwise data fitting procedure on the real part of the corrected signal. The fractions of saturated (SFA), monounsaturated (MUFA), and polyunsaturated (PUFA) fatty acids were finally obtained from ndb and nmidb (14).

### Liver and adipose tissue segmentation

An automated segmentation algorithm was implemented to separately compute quantitative metric abdominal volumes of subcutaneous adipose tissue (SAT) and visceral adipose tissue (VAT). Briefly, the 3D PDFF map was thresholded (t = 50%) to build adipose and non-adipose tissue 3D masks. From the center of the 3D adipose tissue mask, a morphological mathematical operation was performed to initiate a localized active contour (15). The latter was employed to delimitate the SAT inner border (or VAT outer border) and was next propagated to adjacent slices with a 7-pixel length square window, a regularization term equal to 1,300 iterations, and the epsilon coefficient for the Heaviside function was set to 1 (see Supplementary Figure S1 for an illustration).

Results from the automatic inline liver segmentation were checked and manually corrected, if necessary, to ensure adequate liver volume measurements. The liver was also manually segmented from the PDFF map using an in-house tool developed in MATLAB R2022b (The MathWorks Inc., Natick, MA, USA) by an experimented physicist blinded to any clinical data (B. L, 15-year experience in MR). Segmentation was performed in a three-dimensional slice-by-slice manner while taking care to avoid large vessels (hepatic veins, portal trunk, and biliary ducts). Steatosis grading used to quantify liver fat was based on PDFF-specific thresholds (S0 < 3.5%, 3.5 ≤ S1 < 15.5%, S2-S3 ≥ 15.5%) to account for differences between histology and PDFF-based measures as proposed recently by Quadri et al. (16) and Park et al. (17) with contemporary CSE-MRI PDFF measurement methods. A segmentation mask was then applied to the T_2_* map. Means and standard deviations were computed from each parametric map.

### Statistical analysis

Statistical power was estimated based on preliminary results (18, 19) and longitudinal studies (20). A minimum of 15 subjects were required to detect changes over time points, assuming *α* = 0.05, power (1-*β*) = 0.95, and a bilateral hypothesis for differences between groups and time points (Stata 18). A total of 32 subjects were recruited to account for drop-offs and maximize subgroup comparisons (sex-specific effects). No drop-off occurred.

All variables were screened for normality using the Shapiro–Wilk test and reported as the mean (SD) or mean [95%CI] when appropriate. To examine the effects of age and sex (between-subjects factors), time (within-subjects factor), and their interactions, we used a 3-way repeated-measures mixed-effects model approach with a compound symmetry covariance matrix and REstricted Maximum Likelihood (REML) fitting. A Geisser–Greenhouse correction was applied to account for the violation of the sphericity assumption. When applicable, pairwise *post hoc* comparisons were performed using Tukey’s test to correct for multiple comparisons. Simple bivariate analysis was used to assess the correlation between liver volume changes, baseline PDFF, and PDFF changes at D + 12. For all analyses, a *p*-value of < 0.05. Statistical analyses were performed using Prism 10.1 (La Jolla, CA, USA) and Stata 18 (College Station, TX, USA).

## Results

Anthropometric data at baseline and the changes are provided in Table 1. As expected, fasting led to a significant weight decrease (*p* < 0.001), with an average weight loss from baseline of −5.9 [5.2; 6.6] kg at D + 12, remaining significant at M + 4 (−2.8 [0.4, 5.1] kg; *p* = 0.015) (Table 1).

**Table 1:** Anthropometric data at baseline and evolution at each time points of the fasting protocol.

Parameters	Patients sub-categories	D-1	D + 12	M + 1	M + 12
Weight (kg)	All (*N* = 32)	78.8 (13.1)	72.9 (12.2)***	74.7 (12.2)***	76.1 (13)*
	Aged (*N* = 16, >50yo)	79.98 (11.13)	73.96 (10.52)***	74.53 (9.91)***	74.29 (9.07)*
	Young (*N* = 16 < 50yo)	77.74 (14.71)	71.92 (13.60)***	74.48(13.92)***	77.81 (16.12)
	Female (*N* = 16)	72.78 (9.71)	67.73 (9.12)***	68.74 (8.50)***	70.56 (10.20)*
	Male (*N* = 16)	85.24 (13.11)	78.41 (12.59)***	80.63 (12.24)***	81.54 (13.43)
Height (cm)	Aged	172.3 (8.5)			
	Young	175.18 (9.3)			
	Female	167.18 (5.3)			
	Male	180.88 (5.8)			
BMI (kg/m^2^)	Aged	26.92 (3.75)	24.89 (3.54)***	25.08 (3.33)***	25.00 (3.05)*
	Young	26.16 (4.95)	24.21 (4.95)***	25.07 (4.68)***	24.65 (8.24)*
	Female	24.50 (3.27)	22.79 (3.27)***	23.13 (2.86)***	22.35 (6.65)*
	Male	28.69 (4.41)	26.39 (4.41)***	27.14 (4.12)*	27.44 (4.52)*
Age (years)	Aged (>50 y)	58.75 (6.09)			
	Young (<50 y)	38.12 (6.39)			
	Female	48.53 (13.48)			
	Male	47.69 (10.97)			

Legend for *post-hoc* comparisons: D + 12, M + 1 &M + 4 vs D-1: *p < 0.05; **p < 0.01; ***p < 0.001.

### Liver volume and fat content changes in all subjects

**Figure 1:**
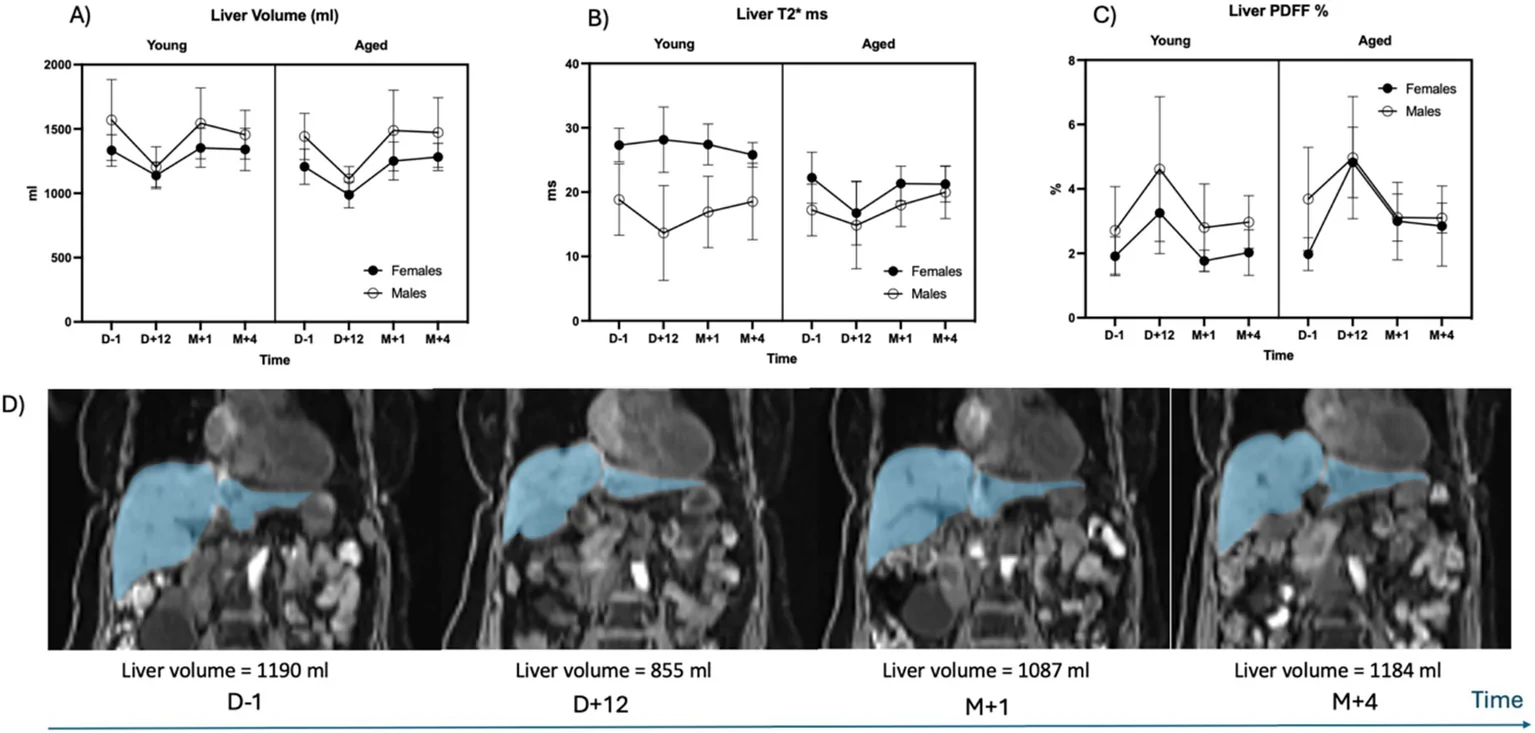
**(A)** Liver volume, **(B)** apparent relaxation time T2* and **(C)** PDFF significant variations during the fasting intervention, with sex-related differences and age-related effects showing different pattern of time changes by quantitative in sub-groups. **(D)** Sample segmentation results in the water images **(D)** showing the segmented liver (blue) for a female GENESIS participant over time.

Table 2 summarizes all quantitative MRI metrics (PDFF, T2*, and fatty acid composition) as well as the volumes of abdominal adipose tissues for each time point and group, also indicating statistical significance. The values denote the average (standard deviation). Globally, liver volume decreased during the fasting intervention (*p* < 0.0001), with sex-related differences (*p* = 0.017), but no age-related effects (Figures 1A,D). The average decrease in liver volume was −268 [−336; −200] ml, corresponding to a − 20.9% relative volume change. At D + 12, liver volume loss was higher in males (−348 [−470, −226] ml, a − 25% average volume change) than females (−206 [−268; −144] ml, a − 16% average volume change). Liver volumes returned to baseline values at M + 1 (*p* = 0.87) and M + 4 (*p* = 0.99).

**Table 2:** MRI metrics (PDFF, T2* and fatty acid composition) and the volumes of abdominal adipose tissues and liver for each time point.

	**Variables**	**Time**					**Sex**						**Age**						**Interactions**		
																			Time*sex	Time*age	Sex*age
		**D-1**	**D+12**	**M+1**	**M+4**	***p***		**D-1**	**D+12**	**M+1**	**M+4**	***p***		**D-1**	**D+12**	**M+1**	**M+4**	***p***	***p***	***p***	***p***
	Liver volume (ml)	1333.2 (159.5)	1108.5 (149.3)****	1397.1 (255.7)	1379.4 (202.2)	<0.0001	Male	1510.5 (250.0)	1162.1 (147.1)	1518.0 (258)	1463.2 (197.6)	0.017	Young	1436.5 (272.7)	1167.5 (148.7)	1436.4 (256.4)	1394.5 (201.7)	0.197	0.024	0.196	0.7164
							Female	1273.6 (169.3)*	1067.5 (146.1)	1304.7 (188.7)*	1311.4 (162)		Old	1307.4 (200.9)	1041.0 (122.6)	1352.2 (256.8)	1363.4 (209.1)				
	Liver T2* (ms)	21.8 (5.9)	19.1 (8.8)**	21.5 (5.7)	21.5 (4.9)	0.0009	Male	18.0 (6.0)	14.3 (8.9)	17.5 (5.8)	19.3 (5.0)	0.0004	Young	23.6 (6.3)	22.4 (9.8)	23.2 (6.9)	22.4 (5.9)	0.050	0.141	0.060	0.033
							Female	24.9 (4.7)***	22.8 (8.5)**	24.6 (4.8)***	23.5 (3.6)*		Old	19.9 (5.1)	15.9 (6.4)*	19.8 (3.7)	20.7 (3.8)				
	Liver PDFF (%)	2.5 (1.3)	4.3 (1.8)****	2.6 (1.1)	2.7 (1.1)	<0.0001	Male	3.2 (1.4)	4.8 (1.8)	3.0 (1.4)	3.0 (1.0)	0.024	Young	2.3 (1.2)	3.8 (1.9)	2.2 (1.0)	2.5 (1.0)	0.047	0.578	0.764	0.4741
							Female	1.9 (0.7)*	4.0 (1.7)	2.3 (1.2)	2.4 (1.2)		Old	2.8 (1.5)	4.9 (1.6)	3.1 (1.1)*	3.0 (1.1)				
**Abdo SAT**	T2* (ms)	21.0 (3.4)	22.0 (3.3)***	19.0 (3.5)***	19.6 (3.2)	<0.0001	Male	19.8 (3.5)	18.5 (3.5)	17.4 (3.5)	18.3 (3.3)	0.047	Young	19.76 (3.74)	19.36 (3.45)	17.90 (3.90)	18.72 (3.44)	0.012	0.094	0.392	0.738
							Female	22.1 (2.9)*	21.3 (2.6)**	20.6 (2.9)**	20.9 (2.6)**		Old	22.34 (2.39)*	20.67 (3.13)	20.34 (2.73)*	20.44 (2.83)				
	PDFF (%)	90.0 (2.3)	89.4 (2.4)***	88.6 (2.5)****	89.2 (2)*	<0.0001	Male	88.8 (2.4)	88.2 (2.4)	87.3 (2.3)	88.1 (1.7)	<0.0001	Young	89.2 (2.6)	88.6 (2.5)	87.8 (2.6)	88.7 (1.9)	0.007	0.226	0.105	0.9216
							Female	91.0 (1.7)**	90.4 (2.0)**	89.8 (2.0)***	90.2 (1.7)***		Old	90.8 (1.7)	90.1 (2.2)*	89.4 (2.2)	89.6 (2.0)				
	SFA (%)	58.5 (2.5)	58.3 (1.9)	58.5 (3.1)	58.9 (2.6)	0.088	Male	58.3 (3.2)	58.1 (2.2)	57.9 (4.1)	58.8 (3.2)	0.413	Young	58.5 (2.9)	58.7 (1.8)	58.3 (4.2)	58.8 (3.3)	0.812	0.267	0.820	0.363
							Female	58.7 (1.6)	58.6 (1.6)	59.0 (1.8)	59.0 (1.9)		Old	58.5 (2.0)	58.0 (2.0)	58.6 (1.6)	59.0 (1.8)				
	MUFA (%)	30.1 (2.4)	29.8 (2.3)	29.1 (2.7)	29.5 (2.2)	<0.0001	Male	29.0 (2.6)	28.7 (2.4)	27.5 (2.7)	28.3 (2.1)	<0.0001	Young	29.5 (2.7)	29.1 (2.4)	28.3 (2.9)	28.7 (2.4)	0.019	0.015	0.495	0.452
							Female	31.2 (1.7)*	30.8 (1.8)**	30.6 (1.8)***	30.7 (1.5)***		Old	30.8 (1.9)	30.5 (2.1)	30.0 (2.3)	30.2 (1.8)				
	PUFA (%)	11.4 (3.4)	11.9 (2.8)**	12.4 (4.7)**	11.6 (3.6)	0.0030	Male	12.7 (4.3)	13.3 (3.3)	14.6 (6.0)	13.0 (4.6)	0.0052	Young	11.9 (4.2)	12.2 (2.9)	13.4 (6.2)	12.5 (4.7)	0.118	0.023	0.691	0.256
							Female	10.1 (1.4)	10.6 (1.4)**	10.5 (1.5)*	10.3 (1.4)*		Old	10.7 (2.0)	11.5 (2.8)	11.4 (2.2)	10.8 (1.9)				
	Volume (cm^3^)	2706 (1190)	2578 (1103)	2317 (1106)	2405 (1224)	0.0004	Male	2215.9 (1029.7)	2091.6 (879.1)	1902.8 (978.0)	1923.4 (1020.7)	0.0007	Young	2367.4 (1171.0)	2254.2 (1060.7)	2051.2 (1190.6)	2253.3 (1342.6)	0.011	0.5643	0.1791	0.5483
							Female	3167.2 (1171.8)**	3008.2 (1122.9)**	2683.2 (1108.4)**	2885.6 (1249.7)**		Old	3065.7 (1136.5)*	2902.9 (1078.4)*	2583.6 (978.8)	2555.7 (1116.3)				
**Abdo VAT**	T2* (ms)	20.0 (3.0)	18.9 (2.4)***	19.0 (2.8)***	19.0 (2.7)	0.0023	Male	21.0 (3.0)	19.8 (3.3)	19.8 (3.2)	19.4 (3.0)	0.278	Young	18.4 (2.4)	17.5 (2.3)	17.8 (2.4)	18.1 (2.4)	0.0016	0.193	0.278	0.407
							Female	18.9 (2.7)	18.1 (2.6)	18.4 (2.4)	18.5 (2.2)		Old	21.6 (2.8)**	20.4 (3.0)**	20.3 (2.7)**	19.9 (2.7)*				
	PDFF (%)	84.6 (2.4)	84.1 (2.7)	83.7 (2.3)	83.8 (2.0)	<0.0001	Male	85.4 (2.7)	84.9 (3.1)	84.5 (2.7)	84.4 (2.2)	0.168	Young	83.4 (1.8)	82.8 (2.1)	82.7 (1.8)	83.2 (1.8)	0.0015	0.599	0.0013	0.575
							Female	83.8 (1.8)	83.3 (2.0)	83.1 (1.7)	83.2 (1.7)		Old	85.9 (2.3)	85.3 (2.6)	84.8 (2.4)	84.4 (2.1)				
	SFA (%)	60.1 (3.5)	60.0 (3.5)	60.0 (3.7)	60.5 (3.8)	0.638	Male	59.8 (2.8)	59.9 (2.6)	60.3 (3.7)	60.0 (3.8)	0.746	Young	59.8 (4.1)	60.3 (3.6)	60.1 (4.1)	60.3 (4.4)	0.9885	0.602	0.445	0.780
							Female	60.3 (4.1)	60.0 (4.3)	59.8 (3.9)	60.9 (3.7)		Old	60.4 (2.7)	59.6 (3.5)	60.0 (3.4)	60.7 (3.1)				
	MUFA (%)	28.8 (2.1)	28.2(2.2)**	28.3(2.2)**	28.4 (1.8)	0.0008	Male	29.7 (2.2)	28.8 (2.5)	28.5 (2.6)	28.9 (1.7)	0.119	Young	28.0 (1.7)	27.0 (1.8)	27.1 (2.2)	27.7 (1.8)	0.0053	0.002	0.058	0.2964
							Female	27.9 (1.6)*	27.6 (1.7)	27.9 (1.8)	27.8 (1.7)		Old	29.6 (2.2)**	29.4 (1.9)*	29.2 (1.7)	29.0 (1.5)				
	PUFA (%)	11.1 (3.5)	11.8 (3.3)***	11.8 (3.6)***	11.2 (3.7)	0.227	Male	10.5 (2.2)	11.3 (2.3)	11.2 (3.3)	11.0 (4.2)	0.641	Young	12.3 (4.2)	12.7 (3.3)	12.8 (4.1)	12.0 (4.2)	0.124	0.479	0.900	0.795
							Female	11.9 (4.3)	12.3 (4.0)	12.4 (3.9)	11.3 (3.3)		Old	10.0 (2.1)	11.0 (3.2)	10.8 (3.0)	10.3 (3.1)				
	Volume (cm^3^)	1081 (1037)	982 (1012)***	887 (916)****	911(871)*	0.006	Male	1513.4 (1303.4)	1405.5 (1294.1)	1281.3 (1173.5)	1267.0 (1072.7)	0.0387	Young	651.4 (686.0)	593.1 (735.7)	540.6 (708.0)	648.6 (680.0)	0.0018	0.245	0.0005	0.079
							Female	674.1 (441.1)	608.2 (449.9)	539.3 (380.2)	555.1 (382.8)		Old	1537.5 (1166.3)**	1370.8 (1119.3)**	1233.7 (987.2)**	1173.5 (978.8)*				
	VAT fraction (%)	25.5 (14.4)	23.6 (15.1)***	24.1 (14.5)	24.6 (13.6)	0.022	Male	35.3 (13.8)	33.3 (16.2)	35.0 (13.5)	34.3 (11.6)	<0.0001	Young	19.7 (9.7)	17.6 (10.0)	19.1 (10.1)	21.2 (10.7)	0.0044	0.1251	0.0018	0.0841
							Female	16.2 (6.9)***	15.0 (6.6)**	14.5 (6.3)***	15.0 (6.8)****		Old	31.7 (16.2)*	29.6 (17.1)*	29.2 (16.7)*	28.0 (15.5)				

Values denote the average (standard deviation). Legends for *post-hoc* comparisons: For time variables, comparisons are against D-1 used as control: *p < 0.05; **p < 0.01; ***p < 0.001. For Sex and Age variables, *post-hoc* comparisons were performed for each time point.

Liver composition analyses revealed significant changes in T2*, with an age × sex × time interaction (*p* = 0.035). While T2* was higher in young females and remained unchanged by fasting (D + 12), fasting decreased T2* in all other subgroups (all males and aged females) (*p* = 0.011). Fasting-induced changes in T2* were reversed and returned to baseline values at M + 1 and M + 4 (Figure 1B).

Liver fat content showed significant changes over time (*p* < 0.0001), with a marked 72% increase at D + 12 mean(SD) = 4.3 (1.8) compared to baseline 2.5 (1.3). The PDFF was higher in aged (*p* = 0.047) and male (*p* = 0.024) subjects at all time points. The fat fraction increased in both sexes and in both aged and young subjects at end-fasting (*p* < 0.0001) while returning to baseline values at M + 1 (*p* = 0.92), as illustrated in Figure 1C.

### Liver volume and changes according to liver steatosis stage

**Figure 2:**
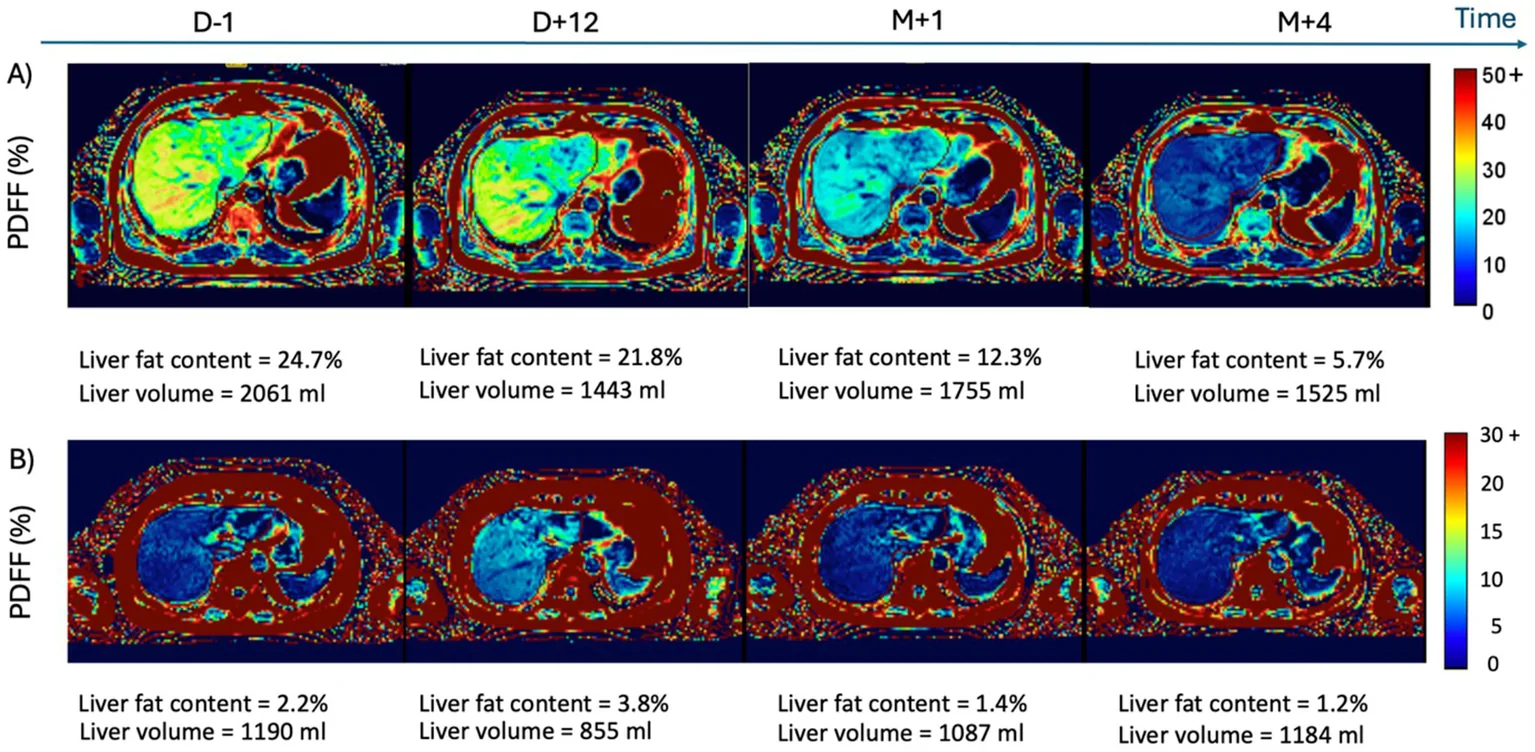
Examples of PDFF map in the abdomen and associated liver volume reduction in a male **(A)** and female **(B)** participants, illustrating the difference in behavior observed other the GENESIS trial depending on the steatosis grade. The male participant with moderate (S2) liver steatosis (PDFF≥15.5%) exhibits in the liver a strong decrease over time and a real benefit of the fasting protocol, switching to S1 grade at M + 1. In the female and S0 subjects, we observed a typical increase in PDFF in the liver at end-fasting (D + 12), returning rapidly to even lower values than baseline M + 1 and M + 4 and illustrative the physiologic response to fasting and adaptive reciprocity of lipid and glucose metabolisms, which switches from one system to another.

While most subjects (23/32) had no steatosis (S0) at baseline PDFF measures before fasting, 7 subjects had mild (S1) liver steatosis (3.5 ≤ PDFF<15.5%) and 2 had moderate (S2) liver steatosis (PDFF≥15.5%). At end-fasting (D + 12), we observed an increase in PDFF in 22/23 S0 subjects and a more balanced behavior in S1 subjects with a PDFF increase in 4/7 subjects, while both S2 subjects showed decreased PDFF values. Illustrative examples of S0 and S2 subject are proposed in Figure 2.

We investigated whether changes in PDFF were a significant contributor to changes in liver volume. After excluding the two S2 outliers, there was no correlation between liver volume reduction at D + 12 and baseline PDFF (r = 0.02, *p* = 0.89) or PDFF change (r = 0.29, *p* = 0.11). Nonetheless, liver volume reduction at D + 12 was higher in S2 subjects (34.6 ± 6.5%) than in S0 (19.1 ± 7.8%) and S1 (17.0 ± 2.6%) subjects.

#### Subcutaneous adipose tissue (SAT)

**Figure 3:**
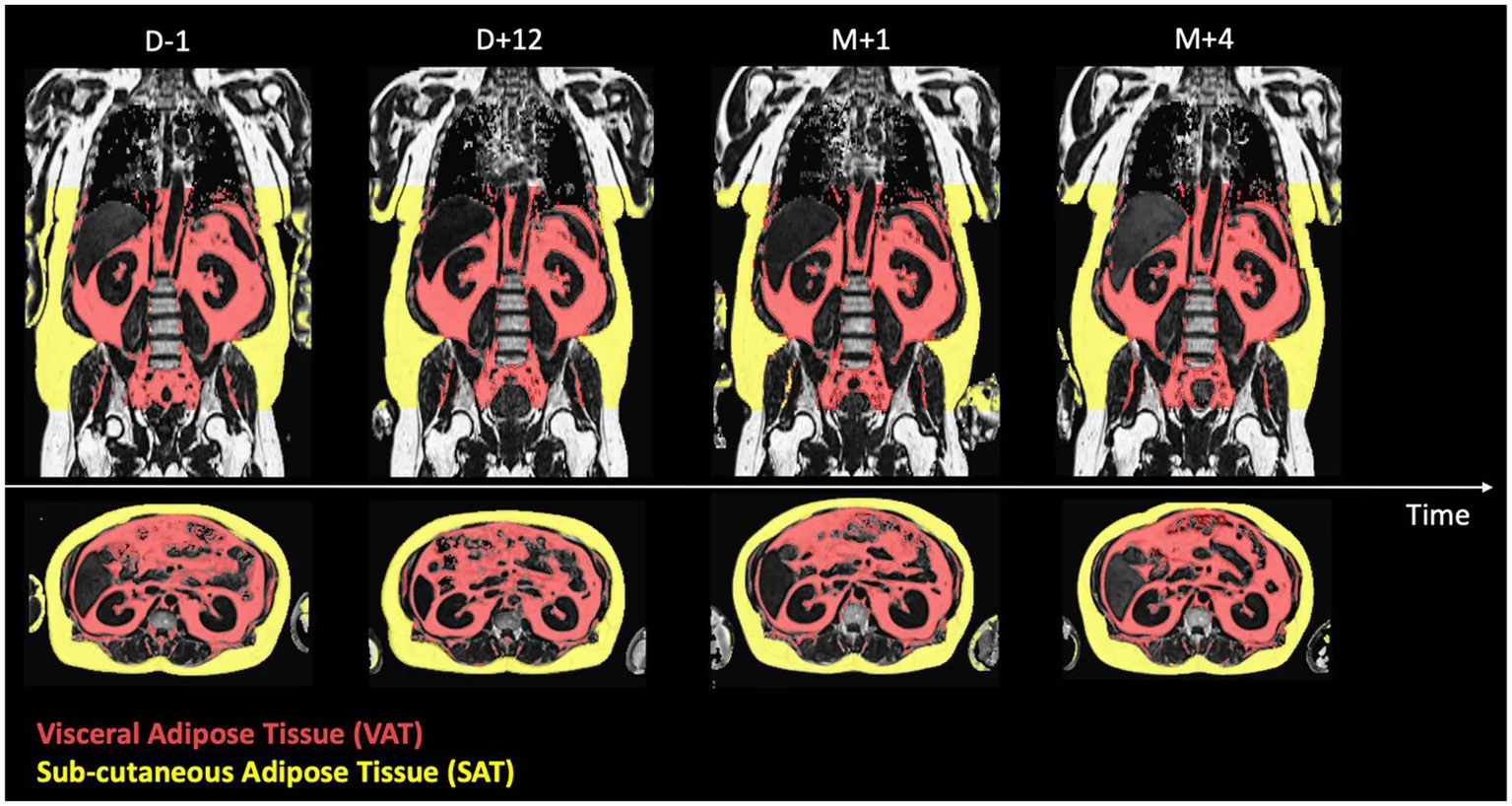
Longitudinal evolution of respective Visceral and Sub-cutaneous Abdominal Adipose Tissue, along GENESIS long-term fasting illustrated in one male subject. (VAT) and (SAT) segmentations are, respectively, displayed in red and yellow overlay on fat only whole body reformatted coronal images.

**Figure 4:**
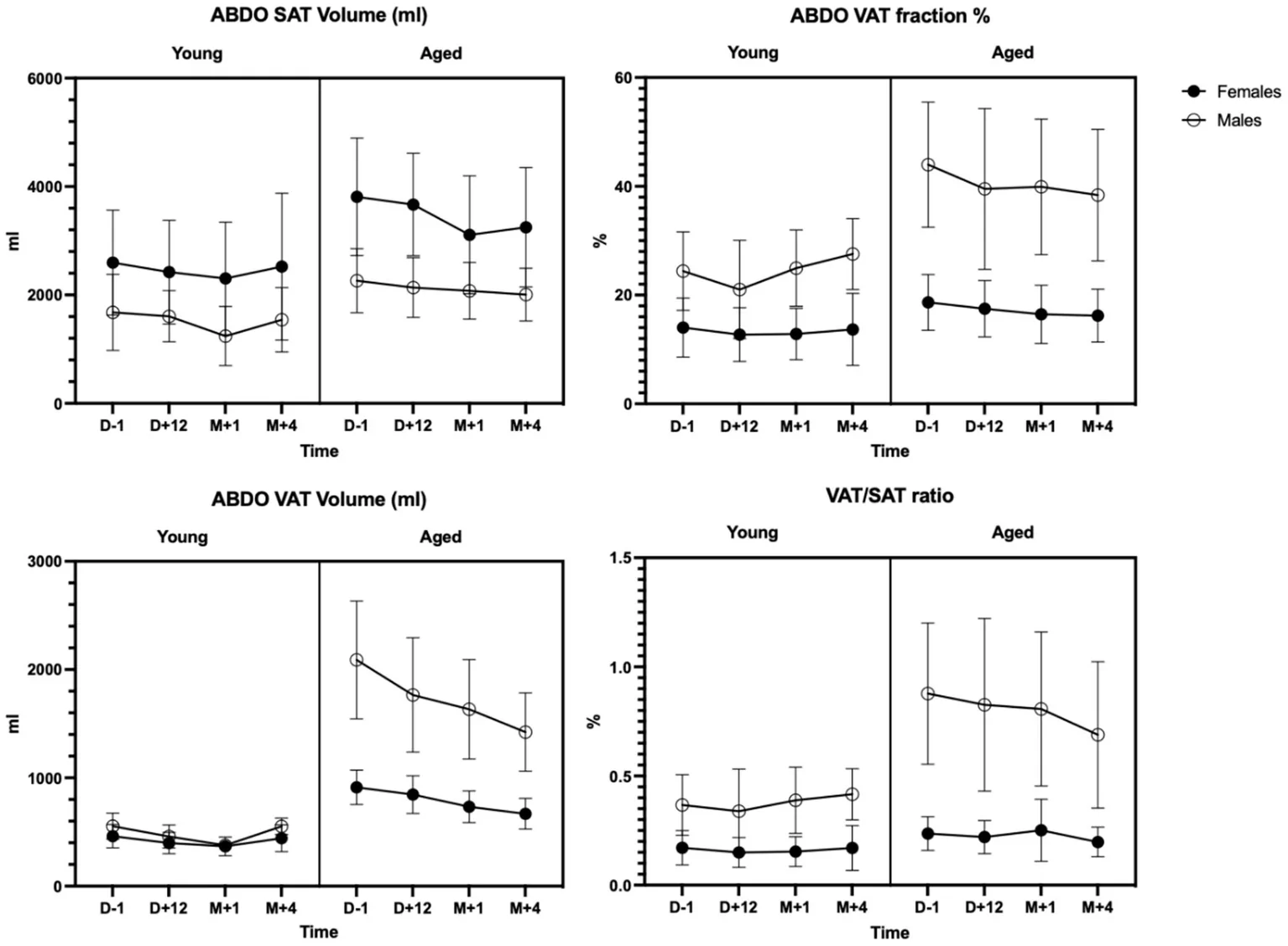
Abdominal SAT (Subcutaneous Adipose Tissue) and VAT (Visceral Adipose Tissue) volume significant variations during the fasting intervention, with sex-related differences and age-related effects showing different pattern of time changes by quantitative in sub-groups.

The SAT volume showed changes over time (*p* = 0.0004), with sex-related differences (higher volumes in females; *p* = 0.01) and age-related differences (higher in aged, *p* = 0.0007) (Figures 3, 4). Although SAT volume was marginally decreased at the end of the fasting period at D + 12 (−102.1 [−210; 5.78] ml, *p* = 0.066), the SAT volume decrease was more pronounced and significant at M + 1 (−373.6 [−588.6; −158.5] ml, *p* = 0.0005) and remained significantly lower at M + 4 (−260.2 [−503.1; −17.4], *p* = 0.033) than at baseline.

SAT T2* decreased significantly over time at all time points compared to baseline (all *p* < 0.002), with lower T2* in males (*p* < 0.001) and in young individuals (*p* = 0.012) without interactions among factors (Supplementary Figure S1). SAT PDFF also decreased significantly over time (*p* < 0.0001). When compared to baseline, PDFF decreased not only at D + 12 (*p* = 0.0003), but also at M + 1 (*p* < 0.0001) and M + 4 (*p* = 0.015). The PDFF was significantly lower in males (*p* < 0.0001) and young subjects (*p* = 0.006). While there were no significant changes in SFA content, MUFA content changes over time were sex-dependent with a time × sex interaction (*p* = 0.014). Larger variations and lower MUFA content were observed in males than females (*p* = 0.019). Time variations in PUFA content depended on sex (time × sex interaction, *p* = 0.023) (see Figure 2).

#### Visceral adipose tissue (VAT)

VAT volume changes over time depending on the age group (time × age interaction effect, *p* = 0.0005) (Figure 4). The VAT volume was nearly stable in young subjects of both sexes. In contrast, it decreased over time and up to M + 4 in the aged subjects, with a more prominent effect in males (*p* = 0.038). T2* was higher in older subjects (*p* = 0.0016). The VAT PDFF decreased over time in aged subjects (time × age interaction, *p* = 0.0013), without significant differences related to sex. Regarding fat composition, while there were no significant changes in SFA and PUFA components, MUFA % decreased over time (*p* = 0.008) and was higher in aged subjects (*p* = 0.005).

## Discussion

Scientific studies investigating fasting have traditionally focused on body weight and body mass index (BMI) as proxies for weight loss-induced health benefits. However, the location of fat accumulation is more critical to its negative health impact than its total amount. To our knowledge, this is the first study to quantify abdominal morphological and metabolic changes following long-term fasting for 12 days in a representative population of healthy subjects. We found that (1) fasting caused reversible adaptive changes in the liver, with a 21% volume reduction and a temporary increase in fat content in subjects with normal (S0) liver composition (Figures 1, 4) variations in abdominal adipose tissue compartments (VAT and SAT) followed different, delayed kinetics. Volume changes in these compartments were detectable at the end of fasting but continued to decrease until the end of the follow-up at M + 4 (Figure 2), especially in the subgroup with the highest VAT values (aged men).

### Liver changes

The 21% reduction in liver volume observed in this study is comparable to the 12–27% decrease achieved with very low-calorie diets (400–800 kcal/day) used preoperatively to reduce bariatric surgery risks (21, 22) and in lean and obese women (23). Although the timing of these changes is not fully understood, they may occur rapidly, as evidenced by a study of eight healthy subjects showing a 17% (range 9–31%) reduction in liver volume following circadian variations (24) and another study by Sedivy et al. after 2 days of fasting in lean and obese women (23). This rapid change is likely because glycogen, which is used for energy production during the first 24 h of fasting, binds 3–4 g of water per gram and constitutes 5–6% of the hepatocyte volume (23, 25). Given that most liver cells are hepatocytes, the observed reduction in liver volume in this study likely reflects physiological variations in glycogen content (23, 26). Additionally, liver volume reduction can result from fat elimination in individuals with severe MAFLD (22). This was observed in two male subjects (males) with S2 steatosis who exhibited the largest liver volume reduction (34.6 ± 5%).

If the steatosis cut-off value to quantify liver fat content is commonly set to 5% in guidelines when relying on histology measures (27–29), steatosis grading is based on PDFF-specific thresholds that have been recently proposed and recommended to account for differences between histology and PDFF-based measures and to prevent misclassification and bias across methods (16, 17), resulting in a higher number of subjects categorized as having steatosis. Our study strengthens the hypothesis of Sedivy et al. that the hepatic response to fasting is strongly related to initial liver fat content. Accumulation of fat in the liver, as a substrate for the production of ketone bodies, seems to occur only in subjects with insufficient liver fat.

The GENESIS study aimed to recruit healthy, representative subjects, and the observed rate (9/32) of liver steatosis aligns with global population averages (30). The liver fat content temporarily increased at the end of fasting in most subjects with S0 and S1. This is likely to be a physiological response to the mobilization of stored fat for energy during fasting. When free fatty acids mobilized from adipose tissue are not used for *β*-oxidation in the liver mitochondria, they can be re-esterified into triglycerides and subsequently deposited in the hepatocyte cytoplasm (31). This fasting-induced phenomenon was observed in mice after 24 h of fasting (32), dogs fasted for 18 h (33), and starved zebrafish larvae (34). Increased hepatic fat content has also been reported previously in a study comparing liver fat response after a short 2 days fasting in lean and obese women (23), with no statistical differences between groups in hepatic volume reduction. It has also been described in human skeletal muscle as early as after 3 days of fasting (35) and after 12 days of fasting (36), with levels returning to baseline after 1 month of refeeding (37), and in the myocardium after a 3-day VLCD (19). In the aforementioned study, the authors reported that after 3 days, hepatic triglyceride levels remained low, suggesting that the increase in liver fat content may occur more progressively and/or be influenced by caloric intake. These findings indicate that the increase in liver fat content is likely a physiological adaptation to fasting metabolism rather than a pathological condition.

In our cohort, while the PDFF increased temporarily during end-fasting, we simultaneously observed a decrease in liver T2*. This drop corresponds to stronger cumulative field homogeneities of lipids and iron. Indeed, water loss induced by the osmotic effect of glycogen depletion may lead to a higher volume fraction of lipid droplets, independent of the impact of larger lipid droplets, along with an increase in the iron concentration (while the amount of iron remains constant). The decrease in T2* observed in most subjects, except young females, is likely related to a higher volume fraction of lipid droplets, leading to a shorter T2*. In younger females, T2* variations are likely to be modulated by iron and estrogen levels (38).

When considering the two S2 subjects, since ectopic lipid accumulation in the liver is directly related to hepatic lipotoxicity, leading to exacerbation of MAFLD to MASH and hepatocarcinoma, this observation suggests that long-term fasting may help prevent MASH. This result is also consistent with a previous study that showed normalization of the fatty liver index following long-term fasting (11). Indeed, CSE-MRI allows for fast, all-in, injection-free quantitative and multi-organ monitoring of dietary interventions, with robust analysis of fat composition, fat adipose distribution, and organ volumes (39, 40). Simultaneous assessment of multiple important fat and organ compartments by MRI could increase the understanding of the complex interplay during fasting. It can be used to quantify the impact of the metabolic switch on fat burning in different organs.

### VAT/SAT changes

The decrease in VAT and SAT volumes not only occurred at the end of fasting, but further decreased up to M + 4 after food reintroduction. This was particularly pronounced in the subgroup of older men with higher VAT and SAT volumes (Figure 4). The VAT/SAT ratio, an independent predictor of Major Adverse Cardiovascular Events (MACE) (41), was positively influenced by a continuous decline of up to M + 4 in these individuals. In both the VAT and SAT compartments, MUFA showed larger variations compared to PUFA and SFA, indicating that they were mobilized during fasting. Mobilization selectivity is linked to the dispersion of TAGs within lipid droplets and depends on their polarity. Shorter and less saturated fatty acids, which are more polar, are situated outside the lipid droplet. This positioning facilitates the targeting of hormone-sensitive lipases, thereby protecting longer-chain fatty acids (42). Preferential mobilization of unsaturated FA is observed in some migratory birds, fasting annually as part of their seasonal cycle, leading to increased transport rates to oxidation and ultimately higher maximum performance levels (1). Our observations in humans regarding hepatic accumulation of fatty acids (FA), reduction of FA in subcutaneous adipose tissue (SAT), and the selective decrease of monounsaturated fatty acids (MUFA)—but not polyunsaturated (PUFA) or saturated fatty acids (SFA)—in abdominal adipose tissue, are consistent with recent targeted lipidomic analyses of rat tissue extracts following a 3-day fasting protocol, as assessed by gas chromatography coupled with flame ionization detection (GC-FID) (43).

The reduction of fat volume, sustainable 4 months after fasting for visceral fat, constitutes a key point for MASH prevention, since visceral fat is associated with hepatic insulin resistance and severity of MASH, even in lean individuals (44).

## Limitations

This study has a few limitations. First, the limited sample size implies that the findings may not be generalizable to a broader population of individuals. In particular, in terms of participant distribution and given the predominance of S0 cases, we acknowledge the limited generalizability of the findings to the broader MASLD population. Nevertheless, the study design and coupling by age and gender features can be considered exploratory, and the results obtained attest to sufficient statistical power, which will be useful in guiding the design of future studies.

The absence of a control group limited our ability to infer causality, making it difficult to separate the effects of fasting from other aspects of the intervention.

As with any nutritional intervention, especially when carried out in a population with a chronic condition, it is legitimate to question the quality of adherence among the target population, which has been shown to be excellent when the intervention is carried out and monitored in a specialized medical environment (45).

## Conclusion

Our data suggest that hepatic and abdominal responses to long-term 12 days prolonged fasting are dependent on the initial liver fat content. Accumulation of fat in the liver, as a substrate for the production of ketone bodies, occurs independently of the sex and age of all subjects with initial PDFF <3.5%, with more balanced behavior in S1 subjects and clear fat reduction in S2 subjects, where liver fat seems to be sufficient for ketone body production. This study highlights the potential role of MRI in evaluating non-pharmacological interventions for liver health, and demonstrates that the studied fasting program could be a widely applicable and safe non-pharmacological intervention in clinical and research settings to prevent and treat metabolic syndrome. Indeed, case results obtained in subjects with moderate to severe MASH clearly indicated a sustainable reduction of visceral fat, suggesting that fasting could help prevent MASH by rapidly reducing liver fat overload.

## Data Availability

The datasets presented in this article are not readily available but data can be made available upon collaboration and by request to the corresponding author. Requests to access the datasets should be directed to magalie.viallon@creatis.insa-lyon.fr.

## In memoriam

This article is dedicated to the memory of Dr. Françoise Wilhelmi de Toledo, whose lifelong commitment to the study of therapeutic fasting made this work possible. She devoted her career to establishing long-term fasting as a scientifically grounded medical intervention and was a driving force behind the GENESIS research programme. This study reflects what she considered most important: understanding and demonstrating, with modern scientific and highly objective methods, through external collaborations offering rigorous expertise and critical thinkers, how using the unique physiological function of fasting in a medical programme reshapes human physiology and improves metabolic health. Beyond her scientific achievements, Françoise inspired the scientific community through her vision, her curiosity, generosity, and commitment to excellence. Her enthusiasm for advancing knowledge was matched only by her dedication to translating science into better patient care. Although she did not have the opportunity to see the completion of this work, her vision is reflected throughout these pages. We dedicate this publication to her memory with deep gratitude and admiration.
