# Supplementary material for: Impact of long-term fasting on liver and abdominal adipose tissues monitored by quantitative chemical-shift encoded MRI
Source: Front Nutr. 2026 Sep 7;13:1868854. doi: 10.3389/fnut.2026.1868854 (PMC13591618; doi:10.3389/fnut.2026.1868854)
Supplement: Supplementary file 1 [file Data_Sheet_1.zip › Supplementary Images 1 and 2.docx]

**Figure S1** Segmentation of PDFF map in abdomen and pelvis for the extraction of MRI metrics and volumes measurement for the non-adipose tissue (blue volume), subcutaneous (yellow volume) and visceral fat (red volume).

**Figure S2**: Whole-body multi-planar reconstruction of fat (A) and water (B) volumes from the Dixon VIBE-based neck to knee MR-protocol for a female GENESIS participant showing the changes over time. Note the very visible liver reduction on water images (bottom row) and evident thigh subcutaneous adipose tissue (SAT) melting on the fat images (top row).
