# Supplementary material for: Impact of long-term fasting on liver and abdominal adipose tissues monitored by quantitative chemical-shift encoded MRI
Source: Front Nutr. 2026 Sep 7;13:1868854. doi: 10.3389/fnut.2026.1868854 (PMC13591618; doi:10.3389/fnut.2026.1868854)
Supplement: Supplementary file 1 [file Data_Sheet_1.zip › Supplementary Table 1 to 3.docx]

**Supplementary Materials**

**Table S1. Eligibility criteria**

| **Inclusion criteria** | **Exclusion criteria** |
| --- | --- |
| - men and women - age between 20 – 75 years - BMI between 22 – 35 kg/m² - negative COVID-19 test - available written declaration of consent | - intake of medication (cardiovascular diseases, lipid and glucose metabolism) - chronic manifest psychical and psychiatric diseases - participation in another study - pregnancy or breastfeeding - active uncontrolled gastrointestinal disorders including ulcerative colitis, Crohn’s disease, indeterminate colitis, severe irritable bowel syndrome, persistent infectious gastroenteritis, persistent or chronic diarrhea of unknown etiology, recurrent *Clostridium difficile* infection - major surgery of the gastrointestinal tract, in the past five years. Any major bowel resection at any time - intake of antibiotics in the last 2 months - in the MRI subgroup, any MRI contraindication (claustrophobia, pacemakers, MR-incompatible prosthetic valves, metallic implants, foreign metallic body) |

**Table S2:** Summary of the 6-minute Dixon VIBE-based neck to knee body composition MR-protocol presented per region. Initial iso-centering was performed with the laser crosshair on the participant’s clavicles prior acquisition of multi-step T1-weighted VIBE Dixon with a total cranio-caudal coverage of 1.1 m divided over six overlapping slabs of axial 3D spoiled gradient dual-echo images. The acquired images covered a region between the neck and the knees, with some MR parameters differing between the slabs depending on which anatomical region was being imaged. Over the torso region (slabs two to four) imaging was performed during a 20sec expiration breath-hold. The total time for the MR body composition protocol was about 6 minutes.

|  | First slab  (neck region) | slab two to four (torso region) | 5th slab  (Upper leg region) | 6th slab  (lower leg region) |
| --- | --- | --- | --- | --- |
| Repetition Time  TR (msec) | 6.69 | 6.70 | 6.71 | 6.72 |
| Echo time  TE (msec) | 2.39 and 4.77 | 2.39 and 4.78 | 2.39 and 4.79 | 2.39 and 4.80 |
| Slices | 440 | 440 | 440 | 440 |
| Voxel size (mm3) | 64 | 44 | 72 | 64 |
| Matrix size | 224 x 168 | 224 x 174 | 224 x 162 | 224 x156 |
| Breath-hold time (sec) | - | 15 | - | - |
|  |  |  |  |  |

**Table S3:** Main MR parameters of the Breath-hold transverse MRI acquisitions for simultaneous quantification of fat content, fatty acid composition and T2* in the abdomen. The sequence is acquired at the abdominal stage and cover from liver dome to L5-S1, using 3D spoiled-multiple echo gradient echo sequences with a flyback readout gradient. Phase and magnitude images were systematically saved.

|  | Abdominal stage |  |  |  |
| --- | --- | --- | --- | --- |
| Repetition Time TR (msec) | 15.5 |  |  |  |
| Echo time TE (msec) | 12 Echoes : starting from 1.2ms with an echo spacing of 1.2ms (bipolar gradient readout) |  |  |  |
| Slices (Thickness in mm) | 44 |  |  |  |
| Thickness (mm) | 5 |  |  |  |
| Voxel size (mm2) | 64 |  |  |  |
| FOV | 450 x 280 |  |  |  |
| Matrix | 320 x 200 |  |  |  |
| Bandwidth (Hz.pixel-1) | 1565 |  |  |  |
| Breath-hold time (sec) | 2*18sec |  |  |  |
|  |  |  |  |  |
